# Recent advances in understanding psoriasis

**DOI:** 10.12688/f1000research.7927.1

**Published:** 2016-04-28

**Authors:** Franziska C. Eberle, Jürgen Brück, Julia Holstein, Kiyoshi Hirahara, Kamran Ghoreschi

**Affiliations:** 1 Department of Dermatology, University Medical Center, Eberhard Karls University Tübingen, Tübingen, Germany; 2 Department of Immunology, Graduate School of Medicine, Chiba University, Chiba, Japan

**Keywords:** psoriasis, T helper cells, skin inflammation, keratinocyte

## Abstract

T helper (Th) cells producing interleukin (IL)-17, IL-22, and tumor necrosis factor (TNF) form the key T cell population driving psoriasis pathogenesis. They orchestrate the inflammation in the skin that results in the proliferation of keratinocytes and endothelial cells. Besides Th17 cells, other immune cells that are capable of producing IL-17-associated cytokines participate in psoriatic inflammation. Recent advances in psoriasis research improved our understanding of the cellular and molecular players that are involved in Th17 pathology and inflammatory pathways in the skin. The inflammation-driving actions of TNF in psoriasis are already well known and antibodies against TNF are successful in the treatment of Th17-mediated psoriatic skin inflammation. A further key cytokine with potent IL-17-/IL-22-promoting properties is IL-23. Therapeutics directly neutralizing IL-23 or IL-17 itself are now extending the therapeutic spectrum of antipsoriatic agents and further developments are on the way. The enormous progress in psoriasis research allows us to control this Th17-mediated inflammatory skin disease in many patients.

## Introduction

Psoriasis is one of the most common chronic diseases, affecting 2–3% of the adult population and 0.5–1% of children. Due to the frequency of the disease worldwide and its clinical characteristics, psoriasis has gained the interest of many scientists in academia as well as industrial research. The easy accessibility of the skin allows scientists to study cells and mediators in inflamed skin and their relevance in disease pathogenesis in detail. Recent advances in psoriasis pathogenesis improved our understanding of disease mechanisms and resulted in the development of new immunobiologics and small molecules that help to control the chronic inflammation ^1^. Here we summarize the recent findings on the cellular and molecular players that presumably contribute to psoriasis development. In general, psoriasis is considered to be an autoimmune disease and most scientists agree on the central importance of T cells in disease pathogenesis ^2– 4^, yet the psoriatic inflammation may originate from epidermal epithelial cells and innate immune cells. Clearly, a close interaction between mediators and cells of the innate and adaptive immune systems and keratinocytes and endothelial cells is present in psoriasis ^5, 6^.

## Aberrant keratinocyte biology as a pathogenic driver in psoriasis

Decades ago, psoriasis was primarily thought to be caused by aberrant keratinocytes resulting in uncontrolled proliferation of the epidermal cell layers. Early studies on the cellular ‘turnover’ of epidermal cells supported this hypothesis ^7^. The keratinocytes in psoriasis are characterized not only by strong proliferation but also by an altered expression of certain keratins like keratin 16. The concept of altered keratinocytes as pathogenic cells causing psoriasis has gained new attention since different reports published in the beginning of this millennium showed that genetic alterations in epidermal transcription factors can cause skin disorders that resemble human psoriasis clinically and histologically. Mice with altered expression of JunB/c-Jun or phosphorylation of STAT3 in keratinocytes develop skin inflammation with histological and molecular characteristics of psoriasis ^8, 9^. Interestingly, in both models, the psoriatic skin inflammation seems to depend on the presence of immune cells including T cells and their cytokines. In fact, there is a close interaction between cytokines and keratinocytes. A number of cytokines present in psoriatic inflammation promotes keratinocyte proliferation. Intradermal injections of T helper 17 (Th17)-associated cytokines like interleukin (IL)-23 or IL-21 into mouse skin induce epidermal hyperplasia with morphological characteristics of human psoriasis associated with the infiltration of inflammatory T cells. On the other hand, keratinocytes themselves are a cellular source of cytokines. The most famous member is IL-8, a cytokine originally discovered in psoriatic scales ^10^. Another example is the cytokine IL-15, which is expressed in psoriatic epidermis and protects keratinocytes from apoptosis. Interestingly, soluble IL-15Rα has been shown to dampen the psoriatic inflammation by suppressing cytokine secretion from keratinocytes and the expansion of IL-17-producing T cells ^11^. Moreover, keratinocytes are a major source of IL-1 production ^12^. Factors such as cytosolic DNA can trigger inflammasome activation and IL-1 secretion by keratinocytes, which contribute to the psoriatic inflammation ^13^. Other mediators that are linked to psoriasis pathogenesis and that are produced by keratinocytes include antimicrobial peptides like S100A8/9, β-defensins, and cathelicidin (LL-37) ^3^. Taken together, genetic alterations in transcription factors and environmental triggering factors affecting keratinocytes presumably facilitate the manifestation of psoriasis, yet psoriasis pathogenesis seems to be dominated by the activation of immune cells rather than alterations in keratinocytes.

## Central role of immune cells

Several observations support the importance of immune cells in the pathogenesis of psoriasis. One is the transfer of the disease by bone marrow cells. Case reports from individuals undergoing bone marrow transplantation for hematological disorders have linked the disappearance of psoriasis as well as the development of psoriasis in the recipient to the skin status of the donor ^14, 15^. The other observation is that immunosuppressive agents originally introduced for the prevention of organ transplant rejection showed unexpected benefits on the clinical course of psoriasis ^16, 17^. Subsequently, immunosuppressive agents like cyclosporine or methotrexate have been established in the treatment of psoriasis. Genetic data on human leukocyte antigen (HLA) associations as well as data on the presence of oligoclonal T cells in lesional skin and their reactivity towards cutaneous antigens further underline the importance of immune cells in psoriasis pathogenesis. Putative autoantigens in psoriasis include keratins, heat shock proteins, the antimicrobial peptide LL37, and the melanocytic antigen ADAMTS-like protein 5 (ADAMTSL5) ^18– 20^. The recent discovery of ADAMTSL5 as a potential autoantigen in psoriasis is a key finding. The recognition of this protein is restricted to epidermal CD8 ^+^ T cells of patients with psoriasis and a HLA-C*06:02 genotype. Stimulation of ADAMTSL5-specific CD8 ^+^ T cells results in IL-17A production ^20^. Of note, HLA-C*06:02 is known as the HLA locus with the strongest genetic association with psoriasis. In addition to the linkage to certain HLA genotypes, recent investigations revealed that psoriasis is also linked to polymorphisms in genes encoding certain cytokines, cytokine receptors, and transcription factors. Today, there is a widely accepted consensus that psoriasis is an immune cell-mediated disease.

## A prototypic Th17 disease

**Figure 1.**
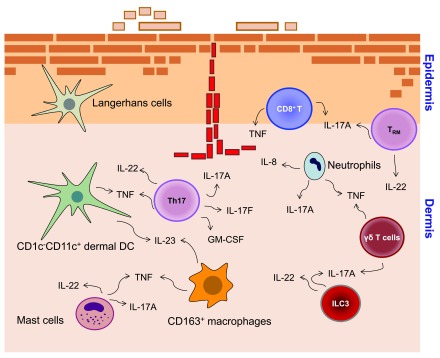
Immune cells and T helper 17 (Th17)-associated cytokines implicated in psoriasis pathogenesis. Characteristic markers and cytokines related to the interleukin (IL)-17/IL-23 immune signature of T cells, dendritic cells (DCs), and associated immune cells in psoriatic skin inflammation.

Among the gene polymorphisms that have been linked to psoriasis are genes encoding *IL23A*, *IL23R*, *STAT3*, *RUNX3*, and *TYK2*. All of these genes are associated with the Th17 immune response ^1^. Th17 cells are characterized by the expression of their lineage-defining cytokine IL-17A. In addition, Th17 cells can produce other cytokines like IL-17F, IL-21, IL-22, tumor necrosis factor (TNF), and granulocyte-macrophage colony-stimulating factor (GM-CSF). Some Th17 populations also secrete IL-9 or IL-10, depending on the signals they receive during initial activation. The differentiation and activation of the Th17 population from naïve T cells depend on cytokines like IL-6, IL-21, IL-1, TGF-β, and IL-23 ^21^. Strikingly, IL-23, its receptor, and its downstream signaling molecule STAT3 are all linked to the genetic susceptibility for developing psoriasis. Of note, the transcription factor STAT3 is also activated by IL-6 and IL-21 and, together with the other Th17-characterizing transcription factor RORγ, STAT3 is responsible for IL-17A and IL-17F expression ^22^. Skin-infiltrating Th17 cells seem to be the central players orchestrating psoriasis pathogenesis ( Figure 1). They interact with tissue cells like keratinocytes and endothelial cells and with various immune cells including dendritic cells (DCs) and neutrophilic granulocytes. The reactivation of memory Th17 cells is presumably responsible for the chronic course of the disease.

## Contribution of skin-resident immune cells

It has become obvious that there is a critical population of memory T cells that resides in the tissue and is involved in the local immune response ^23^. Those specific memory T cells were named “resident-memory T” (T _RM_) cells. T _RM_ cells preferentially reside in epithelial barrier tissues such as the respiratory tract, reproductive tract, and skin ^24– 26^. T _RM_ cells can respond rapidly to pathogenic invaders in the epithelial barrier site, so T _RM_ cells are crucial for the protection of the host from harmful microorganisms. The pathogenic role of T _RM_ cells in immune-mediated diseases including skin diseases like psoriasis is gaining more evidence. A recent study revealed the augmentation of T _RM_ cells in the local inflamed skin of patients with psoriasis ^27^. Moreover, T _RM_ cells in psoriatic skin express higher levels of both *IL17A* and *IL22* compared to those in the skin of healthy individuals. The majority of T _RM_ cells in the epidermis express CD103. T _RM_ cells residing in the dermis show lower expression of this marker ^27^. IL-9-producing T _RM_ cells have also been reported to be present in conditions of skin inflammation like in psoriasis ^28^. Besides T cells, DCs can reside in the skin. DCs are a key population of the immune system, bridging the breaks between innate and adaptive immunity. Among the heterogeneous DC population, CD1c ^-^CD11c ^+^ DCs represent a population of inflammatory dermal DCs. Ultraviolet exposure reduces the number of inflammatory CD1c ^-^CD11c ^+^ dermal DCs in patients with psoriasis ^29^, while the number of CD1c ^+^CD11c ^+^ so-called resident DCs remains unaffected ^30^. A potent marker that allows the discrimination of inflammatory CD1c ^-^CD11c ^+^ DCs from resident CD1c ^+^CD11c ^+^ DCs in patients with psoriasis is TNF-related apoptosis-inducing ligand (TRAIL) ^31^. More intensive studies are needed to identify the environmental signals that induce specific features of T _RM_ cells and resident DCs in the skin under steady state and inflammatory conditions.

## Phenotype of dendritic cells in psoriasis

In general, DCs are a heterogeneous population. In the skin, different types of DCs have been described. The distinct populations are characterized by the expression of certain surface markers and mediators. In psoriasis, certain DC populations like plasmacytoid DCs (pDCs) and dermal myeloid DCs (mDCs) dominate the inflammatory skin, while the number of epidermal Langerhans cells seems to stay stable as compared to non-lesional skin. During initial inflammation, an increased number of pDCs is activated, which results in the release of type I interferon (IFN-α) ^32^. Interestingly, complexes formed by self-DNA or self-RNA and the antimicrobial peptide LL37 have been shown to activate pDCs through Toll-like receptor 9 (TLR9) or TLR7/8, respectively ^33, 34^. Recently, a novel mechanism of pDC activation has been described. As shown for antimicrobial peptides, the Th17-associated cytokine IL-26 can also form complexes with DNA from dying bacterial or host tissue cells and these complexes also promote IFN-α production by pDCs through TLR9 stimulation ^35^. These innate mechanisms seem to be relevant for pDC activation in psoriasis pathogenesis. The activation of pDCs is followed by an increase of CD11c ^+^ mDCs, which express TNF, inducible nitric oxide synthase (iNOS), and IL-23. As mentioned above, inflammatory CD11c ^+^ mDCs do not express CD1c in contrast to skin-resident CD1c ^+^ mDCs. Another DC population that is capable of producing IL-23 is the so-called 6-sulfo LacNAc-expressing population (slanDCs) ^36, 37^. Moreover, CD163 ^+^ macrophages can produce IL-23 ( Figure 1). Taken together, the major function of DCs and macrophages in psoriasis pathogenesis is to provide the signals that promote the Th17 inflammation.

## Non-T cell sources of IL-17A and IL-22 in psoriasis

As we discussed before, the IL-23/IL-17A and IL-23/IL-22 axes play a pivotal role in the pathogenesis of psoriasis ^38^. Besides Th17 cells, IL-17A and/or IL-22 are produced by other types of immune cells including innate lymphoid cells (ILCs) 3, and gamma delta (γδ) T cells ^39– 41^. ILCs have recently been identified as a unique population of innate immune cells that lack antigen-specific receptors. They can be stimulated by cytokines and they produce a series of effector cytokines ^40^. ILCs are now recognized to be divided into three main groups based on the feature of producing lineage-defining cytokines and specific transcription factors ^40, 42, 43^. Among these groups of ILCs, ILC3 including lymphoid tissue inducer (LTi) cells are characterized by the production of IL-17A and/or IL-22 accompanied with high expression of Rorγt ^40, 44, 45^. In the case of humans, ILC3 can be distinguished into several subpopulations based on expression patterns of natural killer (NK) cell markers like NKp44 and NKp46 ^46^. Among these subpopulations, NKp44 ^+^ ILC3 were reported to contribute to the pathogenesis of psoriasis, since IL-17A- and IL-22-producing NKp44 ^+^ ILC3 were increased in both the peripheral blood and the skin of patients with psoriasis ^47^. The crucial role of ILC3 subpopulations in psoriasis pathogenesis is supported by the finding that Rorγt ^+^CD56 ^+^ ILC3, which are capable of producing IL-22, are highly accumulated in the skin of patients with psoriasis ^48^. Another cellular source of IL-17A in the skin is the γδ T cell population ^49^. The majority of dermal γδ T cells express a T cell receptor (TCR) containing Vγ4 together with the chemokine receptor CCR6 ^50^. In an experimental model of psoriasis-like inflammation in mice using the TLR7-agonist imiquimod, dermal Vγ4 ^+^ γδ T cells persist in the skin and contribute to skin inflammation by producing IL-17A and IL-17F ^51, 52^. Consistent with these findings, the increased number of γδ T cells, which produce large amounts of IL-17A, was detected in the affected skin of patients with psoriasis ^53^. More recently, mast cells have also been reported as producers of IL-17A and IL-22 in psoriasis ^54^. Similarly, neutrophils have been suggested as a further cellular source of IL-17A and IL-22. Of note, all immune cells mentioned also produce TNF, a factor well established in psoriasis pathogenesis and treatment. Taken together, various types of immune cells produce the psoriasis-driving cytokines TNF, IL-17A, and IL-22 ( Figure 1).

## Immunotherapies supporting the role of TNF and IL-17A in psoriasis

Based on the immunopathogenesis, antipsoriatic therapies target antigen-presenting cells (APCs), T cells, or their cytokines ( Table 1). Modern small molecules like dimethyl fumarate and the PDE4 inhibitor apremilast both primarily act on APCs. By interfering with intracellular signaling pathways like NRF2 or second messengers like cAMP, they impair the production of pro-inflammatory DC cytokines like IL-23 and in contrast induce the release of anti-inflammatory IL-10. Since they also inhibit IL-12 and TNF production by APCs, dimethyl fumarate and apremilast treatment both result in the suppression of Th17 and Th1 responses ^55, 56^. Thus, silencing IL-23 expression by DCs by small molecules or by RNA interference (RNAi) technology, as recently tested in preclinical settings of autoimmune disease, is an attractive approach ^57, 58^. A new class of modern immunosuppressants is the class of JAK inhibitors ^59^. These compounds interfere with the signaling pathways of numerous cytokines and hormones. Selective JAK inhibitors inhibit the activation and differentiation of multiple Th cell subsets, but they also inhibit the effects of cytokines on non-T cells and non-immune cells ^60^. Thus, the mode of action of all of these compounds is not restricted to APCs and T cells. Although they may also affect other immune cells and tissue cells, they underline the importance of cytokine signaling in psoriasis ^61^. To improve our understanding of psoriasis pathogenesis, it is more helpful to focus on therapeutics targeting single cytokines.

**Table 1.** Modern immunotherapies targeting interleukin (IL)-17/IL-23. The table summarizes approved therapeutics and compounds that are in advanced stage development (according to www.clinicaltrials.gov) and some of their effects on the Th17 response. cAMP, cyclic adenosine monophosphate; DC, dendritic cell; HO-1, heme oxygenase-1; IL, interleukin; NRF2, nuclear factor (erythroid-derived 2)-like 2; PKC, protein kinase C; Th1, T helper type 1; Th2, T helper type 2; Th17, T helper type 17; TNF, tumor necrosis factor.

Immune-modifying category	Immunotherapeutic	Target(s)	Effect on the Th17 response
Small molecules with intracellular mode of action	Apremilast	cAMP/PKC signaling in DCs & macrophages	Inhibits IL-23 and TNF production, induces IL-10
	Dimethyl fumarate	NRF2/HO-1 signaling in DCs & macrophages	Inhibits IL-23 production, induces IL-10, promotes IL-4-producing Th2 cells
	Tofacitinib Ruxolitinib Baricitinib	JAK1/JAK3 JAK1/JAK2 JAK1/JAK2	Inhibit signaling by IL-22 and Th17- promoting cytokines IL-6, IL-21, and IL-23
Anti-cytokine antibodies and fusion proteins	Etanercept Adalimumab Infliximab Golimumab Certolizumab pegol	TNF/lymphotoxin TNF TNF TNF TNF	Impair DC activation and IL-23 production
	Ustekinumab	IL-12/IL-23p40	Impairs Th17 and Th1 responses
	Tildrakizumab Guselkumab BI 655066	IL-23p19 IL-23p19 IL-23p19	Impair Th17 responses
	Secukinumab Ixekizumab	IL-17A IL-17A	Inhibit effects of IL-17A
Anti-receptor antibody	Brodalumab	IL-17RA	Inhibits IL-17A and IL-17F signaling

The first generation of antipsoriatic biologics targeting cytokines focused on TNF. These immunotherapeutics are highly effective in the treatment of psoriasis of skin and joints since they neutralize the effects of TNF on multiple cell types. In psoriatic skin, where TNF is mainly produced by DCs and macrophages, the neutralization of this cytokine rapidly decreases the expression of the Th17-promoting IL-23p40 and some other mediators ^62, 63^. This initial action of TNF neutralization on IL-23 in the skin is followed by the reduction of IL-17A, IL-22, IFN-γ, and TNF. The second generation of anti-psoriatic biologics targeting cytokines focuses directly on the Th17 cytokines IL-23 and IL-17A. The neutralization of p40, a cytokine unit shared by IL-23 and IL-12, is also effective in the treatment of psoriasis and psoriatic arthritis and directly interferes with the activation of Th17 as well as Th1 cells. Importantly, selective inhibition of the IL-23 unit p19 also improves psoriasis. Currently, three antibodies targeting p19 are in phase 3 development for the treatment of psoriasis. Neutralization of IL-23 results in decreased numbers of skin-infiltrating T cells, mDCs, pDCs, and neutrophils, while epidermal Langerhans cells remain unaffected ^64^. Finally, IL-17A itself became a therapeutic target in psoriasis. The first monoclonal antibody directed against IL-17A is already approved for the treatment of psoriasis and psoriatic arthritis ^65^. A second anti-IL-17A antibody recently received a positive opinion by the European Medicines Agency ^66^. Systemic neutralization of IL-17A lowers the expression of IL-17A, IL-17F, IL-22, TNF, IL-6, IL-8, and p40 in the skin ^65^. Of note, an antibody blocking the IL-17 receptor A (IL-17RA) is also effective in psoriasis and is in phase 3 development. These new approaches emphasize the significance of the Th17 pathway in psoriasis.

## Remaining questions

Recent findings have helped us to improve our understanding of psoriasis pathogenesis. We now allocate the mechanisms of previously established antipsoriatic treatments to their effects on the Th17 pathway. This is best illustrated for the use of recombinant IL-4 or the small molecule dimethyl fumarate, which both suppress Th17 cell development ^55, 67, 68^. The new generation of antipsoriatic biologics directly targeting IL-23 or IL-17A underlines the central role of these cytokines in psoriasis pathogenesis. Although we have a battery of systemic treatments for our patients, we do not know which patient will respond adequately to a certain drug. There is still a significant number of primary and secondary non-responders. The lack and the loss of response are mainly observed in patients treated with oral therapeutics and TNF antagonists. Possibly, this will be similar in patients receiving modern drugs directly interfering with the Th17 axis. One of the most important future developments is the establishment of testing methods to predict the clinical response to certain targeted therapies using immunobiologics or small molecules. Definition of useful biomarkers may help to identify responders in an early stage. Some markers like IL-8, IL-19, NOS2, or S100 proteins are typically expressed in psoriatic skin ^10, 67, 69– 71^, but ideal biomarkers are still not established. Furthermore, we have to understand why the same body sites can develop psoriatic plaques, even after long periods of remission. One study demonstrates that epidermal CD8 ^+^ T _RM_ and also CD4 ^+^ T cells reside in the skin even after successful treatment and retain their capability of responding rapidly with the production of IL-17A and IL-22, respectively, upon *ex vivo* stimulation ^27^. Another issue to be studied is the exact mechanism that causes the development of paradoxical psoriasis in patients without prior history of psoriasis who receive TNF antagonists for the treatment of inflammatory colitis or rheumatoid arthritis. Unraveling these immunological mechanisms may also help us to understand the different phenotypes of psoriasis. One example is given by recent findings on pustular psoriasis. Genetic studies could link IL-36RN deficiency and CARD14 mutations to the susceptibility for generalized pustular psoriasis ^72, 73^. Thus, interfering with inflammasome activation or IL-1 family cytokines may be of benefit in such patients ^74^. Similarly, it is of interest to understand why certain environmental factors like infections and drugs but also acquired immunodeficiency result in treatment-resistant cases of psoriasis. To complete our understanding of this chronic inflammatory disease affecting a large proportion of our global population, further research in immunology, genetics, epigenetics, microbiology, and molecular biology is needed.

## Abbreviations

DC, dendritic cells; HLA, human leukocyte antigen; IL, interleukin; ILC, innate lymphoid cell; mDC, myeloid dendritic cell; NK cell, natural killer cell; pDC, plasmacytoid dendritic cell; Th cell, T helper cell; TLR, Toll-like receptor; T _RM_ cell, resident-memory T cell.
